# The Phosphate Source Influences Gene Expression and Quality of Mineralization during *In Vitro* Osteogenic Differentiation of Human Mesenchymal Stem Cells

**DOI:** 10.1371/journal.pone.0065943

**Published:** 2013-06-18

**Authors:** Luisa M. Schäck, Sandra Noack, Ramona Winkler, Gesa Wißmann, Peter Behrens, Mathias Wellmann, Michael Jagodzinski, Christian Krettek, Andrea Hoffmann

**Affiliations:** 1 Trauma Department, Medical School Hannover, Hannover, Germany; 2 Institute for Inorganic Chemistry, Leibniz Universität Hannover, Hannover, Germany; 3 Clinic for Orthopedic Surgery, Medical School Hannover, Hannover, Germany; Georgia Regents University, United States of America

## Abstract

For *in vitro* differentiation of bone marrow-derived mesenchymal stem cells/mesenchymal stromal cells into osteoblasts by 2-dimensional cell culture a variety of protocols have been used and evaluated in the past. Especially the external phosphate source used to induce mineralization varies considerably both in respect to chemical composition and concentration. In light of the recent findings that inorganic phosphate directs gene expression of genes crucial for bone development, the need for a standardized phosphate source in *in vitro* differentiation becomes apparent. We show that chemical composition (inorganic versus organic phosphate origin) and concentration of phosphate supplementation exert a severe impact on the results of gene expression for the genes commonly used as markers for osteoblast formation as well as on the composition of the mineral formed. Specifically, the intensity of gene expression does not necessarily correlate with a high quality mineralized matrix. Our study demonstrates advantages of using inorganic phosphate instead of β-glycerophosphate and propose colorimetric quantification methods for calcium and phosphate ions as cost- and time-effective alternatives to X-ray diffraction and Fourier-transform infrared spectroscopy for determination of the calcium phosphate ratio and concentration of mineral matrix formed under *in vitro*-conditions. We critically discuss the different assays used to assess *in vitro* bone formation in respect to specificity and provide a detailed *in vitro* protocol that could help to avoid contradictory results due to variances in experimental design.

## Introduction

Bone marrow-derived mesenchymal stem cells/mesenchymal stromal cells (MSCs) have long been recognized as a powerful source for the study of osteoblast behaviour and the regeneration of bone tissue. In order to fully understand the mechanism of differentiation and exploit the potential of MSCs for bone regeneration it is indispensable to have a reliable and reproducible *in vitro* model for osteogenic differentiation of MSCs to functional osteoblasts.

*In vitro* systems for osteogenesis recapitulate events during direct (desmal or intra-membranous) osteogenic differentiation. During the *in vitro* differentiation into mature osteoblasts the down-regulation of proliferation, secretion of an organic extracellular matrix and mineralization of this matrix by deposition of hydroxyapatite pose important developmental steps. These are accompanied by a stage-specific expression of bone-related genes [1]. In order to accomplish this process, culture media containing varying concentrations of fetal bovine serum (FBS) are supplemented with additives. It is worth to note that many different sources of FBS are used for the cultivation of MSCs, and that not only the source, but even the batch of FBS can influence the cells, resulting in an incalculable variability in experimental design [2], [3]. Another disadvantage of FBS is its xenogenic origin when considering *in vivo* applications in humans [4]. Dexamethasone, a synthetic glucocorticoid which functions as glucocorticoid receptor agonist, is used in most *in vitro* assays to induce osteogenic differentiation through activation of Wnt/beta-catenin signaling [5]. The effects of dexamethasone on cell proliferation, metabolism and differentiation are concentration as well as context-dependent [6]–[8]. For example, its effect on cell proliferation differs between primary and secondary culture and it is thought that two different osteoprogenitor cell populations exist, with only one being dependent on dexamethasone for proliferation and osteogenic differentiation [9]. Ascorbic acid or a more stable derivative like ascorbate-2-phosphate is added to the differentiation medium to permit collagen type I fibril assembly. An external phosphate source, generally inorganic phosphate or hydrolyzable substrates like β-glycerophosphate (βGP), facilitates the mineralization of the extracellular matrix (mainly collagen fibrils) produced by the cells [10]. Most commonly 10 mM of βGP is added to a medium (mainly DMEM or α-MEM are used, but also BGJb, DMEM:F12, RPMI 1640 and others can be found in the literature [11]) already containing inorganic phosphate (P_i_) and calcium ions (Ca^2+^) (1.0 mM inorganic phosphate and 1.8 mM calcium ions in the case of DMEM as used in this study and α-MEM). In the literature 3 mM of sodium dihydrogen phosphate (NaH_2_PO_4_) or a mixture of the sodium phosphate compounds disodium hydrogen phosphate (Na_2_HPO_4_) and NaH_2_PO_4_ buffered to a pH of 7.4 (from here on referred to as Na_x_H_3-x_PO_4_), as well as 3 mM βGP and other concentrations of these two phosphate sources have also been used [5], [10], [12]–[17]. For a more in-depth review of supplements and basal cell culture media for *in vitro* osteogenesis we refer to Hoemann et al. and Boskey et al. [18], [19]. In summary, a considerable diversity has developed not only in respect to protocols used for two-dimensional *in vitro* osteogenesis but also in respect to the methods and methodology of analysis making it difficult to transfer and compare results between different studies.

Routinely, the outcome of *in vitro* osteogenesis is being assessed by the combination of gene expression analysis and either histological staining for calcium and phosphate ions and/or the measurement of tissue non-specific alkaline phosphatase (TNAP) activity. However, more detailed analyses of the mineralized matrix quality by X-ray diffraction (XRD) or Fourier-transform infrared spectroscopy (FT-IR) are often missing.

Gene expression analysis is commonly used to investigate osteogenic differentiation. Early events of osteogenic differentiation include expression of collagen type I and TNAP [7]. IBSP, a bone-specific glycoprotein that is involved in the nucleation of hydroxyapatite [20], and osteopontin constitute the major part of the non-collagenous extracellular matrix of bone. The latter is a potent inhibitor of *de novo* hydroxyapatite formation and is thought to be a negative regulator of mineralization and expressed at later stages of osteogenic differentiation [21]. The function of osteocalcin (BGLAP), a protein that is expressed at late stages of osteoblast differentiation, is still unclear, although there is some evidence that osteocalcin, like osteopontin, is involved in the negative regulation of bone formation [22].

The use of TNAP protein expression as an indication of successful generation of functional osteoblasts alone is not very suitable in several respects. Firstly, the histological staining using a hydrolyzable substrate which yields a colored product is more a proof of TNAP protein expression than of the actual degree of TNAP activity under differentiation conditions. The osteogenic induction medium, which contains potent inhibitors of its enzymatic activity (P_i_ and pyrophosphate (PP_i_)) [21], is removed prior to the staining. Although the measurement of TNAP enzymatic activity is a more powerful method to visualize the differentiation process than TNAP staining, it must be kept in mind that TNAP is also expressed prominently in human adipocytes and has an important function during the adipogenic differentiation of MSCs [23]. Under classical *in vitro* osteogenic conditions, a percentage of cells develops into adipocytes rather than osteoblasts. Lastly, Hoemann et al. have noted that levels of TNAP activity are not proportional to the mineralization process [19].

The expression of the most common marker genes used for the evaluation of *in vitro* osteogenesis can also be up-regulated by other stimuli, and their expression is not limited to functional osteoblasts. For example, osteocalcin was found to be expressed in neurons [24], an adipocyte cell line and even human adipocytes [25], [26], blood platelets and megakaryocytes [27]. There is evidence that osteocalcin plays an important role not only in the maturation of bone, but also in the regulation of human metabolism [26]. This highlights why it is advisable to base the assessment of in vitro osteogenesis not only on mRNA and/or protein expression.

Apart from the analysis of genes and proteins involved in osteogenesis and of the concomitant mineralization, the study of the composition and quality of the mineralized tissue constitutes the second major aspect of *in vitro* osteogenesis. The mineral in bone matrix is made up mostly of carbonated hydroxyapatite, which consists predominantly of calcium and phosphate ions in a ratio of about 1.63 (in human bone) [28]. The most common methods to visualize *in vitro* mineralization are the *von Kossa* and the *Alizarin Red S* staining, with the former being used to detect phosphate and the latter being used to test for calcium ions. A major limitation of these methods is the lack of specificity in respect to the ions detected [29], [30]. Furthermore, the histological staining cannot provide any information about the composition of calcium phosphate precipitates formed: Not only hydroxyapatite, but also all other calcium phosphate compounds lead to positive staining results [29].

Although more sophisticated methods such as XRD, which is considered to be the gold standard method for identifying mineral phases [18], and FT-IR exist to assess the composition of the extracellular matrix formed during *in vitro* osteogenesis, these are not used often. Both XRD and FT-IR are quite technically demanding and time-consuming methods usually not available in the standard laboratory environment. Moreover, large quantities of material are needed making their application difficult for cell culture analysis. Thus, a simpler method to assess the mineral composition of an *in vitro* sample is desirable.

In the present study we have examined the impact of different phosphate sources and concentrations on gene expression as well as on the temporal concentration of inorganic phosphate in the media. Furthermore, we have evaluated the bone-like mineral quality of these different samples via XRD and FT-IR analyses and we have quantified the calcium and phosphate ion contents in the cell layer by colorimetric methods. Calcium is measured by the formation of a colored complex of calcium with o-cresolphthalein complexone the absorption of which at 570/600 nm is proportional to the amount of calcium present [31], [32]. It needs to be noted that the quantification of the calcium content in the cell layer alone does not allow for reliable evaluation of a successful mineralization, since for example apoptotic bodies as they are present in dead cells may act as nucleating structures for calcium crystal formation [33]. It is thus necessary to take the calcium to phosphate ratio into account. Phosphate is assessed based on the absorption of a complex formed between phosphate and malachite green dye with molybdate the absorption of which at 620 nm is proportional to the amount of phosphate. These cost- and time-effective procedures to determine the calcium : phosphate ratio and the amount of mineral matrix formed under *in vitro* conditions were compared to XRD and FT-IR data.

In this study we demonstrate the distinct disadvantages of using βGP as compared to inorganic P_i_ and show that the colorimetric methods for the determination of calcium and phosphate ions are valid alternatives to obtain an idea of the quality of the mineralized matrix. Furthermore, a detailed protocol will be given for osteogenic induction of human mesenchymal stem cells (see Supplementary Information).

## Materials and Methods

### Materials

NaH_2_PO_4_, Na_2_HPO_4_, AgNO_3_, *Alizarin Red S*, tris(hydroxymethyl)aminomethane, ethanol and paraformaldehyde (PFA) were purchased from Carl Roth GmbH, Karlsruhe, Germany. Sodium L-ascorbate-2-phosphate, βGP-pentahydrate, dexamethasone, hydroxyapatite, and gelatin B (from bovine skin) were purchased from Sigma-Aldrich, Taufkirchen, Germany. Cell culture flasks were from Greiner, Frickenhausen, Germany. Dulbecco’s Modified Eagle’s Medium with 1 g/l D-glucose (FG 0415) (DMEM), phosphate-buffered saline (PBS) without Ca^2+^, without Mg^2+^, 10× trypsin-EDTA (0,5%/0,2% (w/v) in 10× PBS, penicillin/streptomycin (10000 U/ml/10000 µg/ml) and 1 M 4-(2-hydroxyethyl)-1-piperazineethanesulfonic acid (HEPES) were purchased from Biochrom, Berlin, Germany. Cross-Linked C-telopeptides of Type I Collagen (CICP) Elisa was purchased from Quidel, San Diego, USA. TriReagent and Real Time PCR reagents were purchased from Life Technologies, Darmstedt, Germany. Revert Aid First Strand cDNA synthesis Kit and DNase I were purchased from Fermentas, St. Leon-Rot, Germany. Human recombinant FGF-2 was purchased from Peprotech GmbH, Hamburg, Germany. Fetal bovine serum (FBS) (Hyclone, South American origin, Lot RWC35891) was purchased from Thermo Fisher Scientific, Waltham, USA. In order to minimize effects of source and batch of FBS, one single batch was used for all experiments. DIPI-500 - QuantiChrom™ Phosphate Assay Kit was purchased from Gentaur, Brussels, Belgium. Calcium (CPC) LiquiColor® Test was purchased from Stanbio, Boerne, USA.

### Ethics Statement

Human tissues (bone marrow aspirates or a bone fragment, respectively) were obtained after approval of the institutional ethical committee of Medical School Hannover. Written informed consent was obtained from all donors. All personal information apart from age and gender was deleted. Bone marrow aspirates were harvested by iliac crest aspiration during routine orthopedic procedures from healthy donors and used for MSC isolation. Small bone fragments were obtained from left-over tissue of one additional donor during hip replacement surgery. These were used for XRD studies.

### Cell Culture

Human mesenchymal stem cells were prepared from human bone marrow aspirates according to modifications of a protocol described elsewhere [34]. Briefly, the aspirate containing heparin was mixed with 3 volumes of PBS and filtered to remove tissue debris. The resulting suspension was carefully layered onto a density gradient of one volume Biocoll (ρ = 1.077 g/ml) and centrifuged for 45 minutes at 1000×g without brakes. The mononuclear cells located at the interface were harvested using a pasteur pipette, washed once in PBS, resuspended in MSC medium and seeded in cell culture flasks at a density of 1.6*10^5^ cells per cm^2^ [11]. The MSC medium consisted of DMEM supplemented with 10% (v/v) FBS (not heat-inactivated), 20 mM HEPES, 1% (100 U/ml/100 µg/ml) penicillin/streptomycin, and 2 ng/ml human recombinant FGF-2 [35]. The cells were cultured at 37°C with 5% CO_2_ at 85% humidity. They were washed in PBS and supplied with fresh medium 24 hours after initial seeding, and subsequently every 3–4 days. The cells were always passaged at a density of around 70% by the use of 0.025% Trypsin-EDTA solution and seeded at a density of 2*10^3^ cells per cm^2^.

### Osteogenic Differentiation

Mesenchymal stem cells from four different human donors were used for the osteogenic differentiation tests. Donor A was a 53-year old female, donor B a 47-year old male, donor C a 50-year old female, and donor D was a 70-year old female. The cells from all donors were used for the osteogenic differentiation tests in passage five of *in vitro* culture. In order to induce osteogenic differentiation the cells from the above described four different donors were seeded in 6-well dishes and cell culture flasks (growth area: 75 cm^2^) coated with 2% gelatin B at a cell density of 5*10^3^ cells per cm^2^ and allowed to grow to confluence. Upon reaching confluence, the MSC medium was replaced by osteogenic medium without FGF-2 and consisting of DMEM with 1 g/l D-glucose, 10% (v/v) FBS, 2% HEPES, 1% penicillin and streptomycin, 100 nM dexamethasone and 50 µM ascorbate-2-phosphate as well as one of the following phosphate sources: 3 mM Na_x_H_3-x_PO_4_ (pH 7.4), 10 mM Na_x_H_3-x_PO_4_ (pH 7.4), 3 mM βGP, 10 mM βGP or no external phosphate source. The medium for the control cells consisted of DMEM with 1 g/l D-glucose, 10% (v/v) FBS, 2.5% HEPES and 1% penicillin and streptomycin. The medium was changed twice a week and prepared weekly. Cells for RNA isolation were harvested on days 7, 14, 21 and 28. Quantification of calcium and phosphate ions from the cell layer, FT-IR and XRD analyses as well as Von Kossa and Alizarin Red S staining were performed at day 28. For the quantification of the phosphate concentration in the differentiation media, the supernatant from all different media was harvested and stored at −20°C at each change of medium. All experiments were performed independently with cells from the four different donors.

### RNA Preparation and Analysis

During osteogenic differentiation, the cell layers from the 4 donors were harvested by incubation with TriReagent at room temperature for 5 minutes and stored at –80°C at least overnight. In addition, MSC samples from all donors (n = 19 including the 4 donors from the differentiation experiments) were collected at passaging during expansion i.e. before reaching confluence. For the study presented here, samples from passage 2 were analyzed for their mRNA expression levels of collagen Ia1, osteocalcin and TNAP. The frozen samples were thawed at room temperature, 100 µl of 1-bromo-3-chloropropane per ml of TriReagent were added and centrifuged for 15 minutes at 20.000×g and 4°C. The supernatant was carefully aspirated, mixed with 500 µl isopropyl alcohol and centrifuged for another 30 minutes. After removal of all fluid and a washing step with 80% ice-cold ethanol, the pellet was dried and subsequently recovered in 20 µl of double distilled water. The RNA concentration and purity was determined by measuring the absorption at 260/280 nm. 1 µg of RNA was used for cDNA synthesis, which was carried out after DNase treatment to remove genomic DNA according to the protocol of the supplier using random primers.

### qRT-PCR

Real time PCR analysis was used to quantify the transcripts of 18S rRNA (Hs9999901_s1), bone sialoprotein (IBSP) (Hs00173720_m1), osteocalcin (BGLAP) (Hs01587814_g1), osteopontin (Spp1) (Hs00959010_m1) and tissue non-specific alkaline phosphatase (TNAP) (Hs00758162_m1). The gene specific assays and the Fast Advanced Mastermix were purchased from Life Technologies.

### Von Kossa Staining

Cells were washed with Tris-buffered saline (TBS), then fixed in 4% (w/v) PFA in TBS for 30 minutes followed by another three washes with TBS. A solution containing 5% (w/v) of silver nitrate (AgNO_3_) was added to the cells and incubated in the dark for 30 minutes. After this, the silver nitrate was removed by three washes with double distilled water. The cells were then stored in TBS in the dark until photographic documentation.

### Alizarin Red S Staining

Cells were washed with TBS, then fixed in 4% (w/v) PFA in TBS for 30 minutes followed by another three washes with TBS. 1% (w/v) of *Alizarin Red S* (pH 9.5) was added to the cells for an incubation period of 30 minutes. Subsequently, the cells were washed several times with double distilled water until no further discoloration of the water occurred. The cells were then stored in TBS until photographic documentation.

### XRD

To obtain sufficient material for the XRD investigations, cells had to be cultured in 75 cm^2^ flasks. They were fixed in 4% (w/v) PFA in TBS for 30 minutes followed by digestion with 1.5 ml 0.025% Trypsin-EDTA solution and incubation at 37°C for 48 hours. After centrifugation the supernatant was removed and the remainder was dried in a vacuum concentrator for three days. For sample preparation, the dried cell residue was fixed between two transparent foils. The X-ray diffraction patterns were measured in transmission using a Stoe StadiP diffractometer operated with Ge(111)-monochromatized Cu*K*a1 radiation with a wavelength of λ = 1.54060 Å.

Human bone fragments were used for comparison. The samples were stored at −20°C and pestled to a fine powder for XRD measurements.

### FT-IR

Cells were fixed in 4% (w/v) PFA in TBS for 30 minutes followed by digestion with 0.025% trypsin-EDTA solution and incubation at 37°C for 48 hours. After centrifugation the supernatant was removed and the remainder was dried in a vacuum concentrator for three days. For FT-IR analysis, the cell residue was pestled with dry KBr to a homogeneous powder. Transparent KBr pellets were pressed and were measured at a Fourier-transforma infrared spectrometer (Tensor 27, BRUKER) in transmission mode.

### Quantification of Calcium and Phosphate Ions

Cells were fixed in 4% (w/v) PFA in TBS for 30 minutes, washed twice in TBS, and harvested in 1 M HCl using a cell scraper and incubated at 37°C for 48 hours. Cell debris was removed by centrifugation and the supernatants were collected and stored at −20°C for the subsequent quantification of calcium and phosphate ions from the cell layer. The quantification of calcium and phosphate ions in the cell layer was carried out using the Calcium (CPC) LiquiColor® Test and the QuantiChrom™ Phosphate Assay respectively. Both tests were carried out according to the protocols provided by the respective manufacturer. The medium supernatants harvested during the differentiation process were also assayed for their phosphate concentration.

### CICP

The MicroVue CICP assay was used to measure cross-linked type I C-terminal collagen propeptide (CICP) in the differentiation media in order to assess the collagen production of the cells during osteogenic differentiation. It was carried out according to the protocol provided by the manufacturer.

### Data Analyses

The underlying IR bands of the FT-IR spectra were deduced by curve-fitting assuming Gaussian bands (performed with PeakFit 4.12 by Seasolve), for which the peaks were derived from the calculated second-derivative spectra (Origin Pro 9.0). The reflections of the XRD pattern were resolved by curve-fitting using Lorentzian fits (performed with PeakFit 4.12 by Seasolve), for which the positions of the reflections were derived from the calculated second-derivative spectra (Origin Pro 9.0). Statistical analyses were performed using GraphPad Prism 5.0 (GraphPad Software Inc.). Nonparametric Kruskal-Wallis test with multiple comparison analysis with p≤0.05 was used to detect significant differences between groups, with p≤0.05 significant = *, 0.01≥p≥0.001 very significant = **, p≤0.0001 highly significant = ***.

## Results

### βGP Causes Non-physiological Fluctuations in the Availability of Free Phosphate

**Figure 1:**
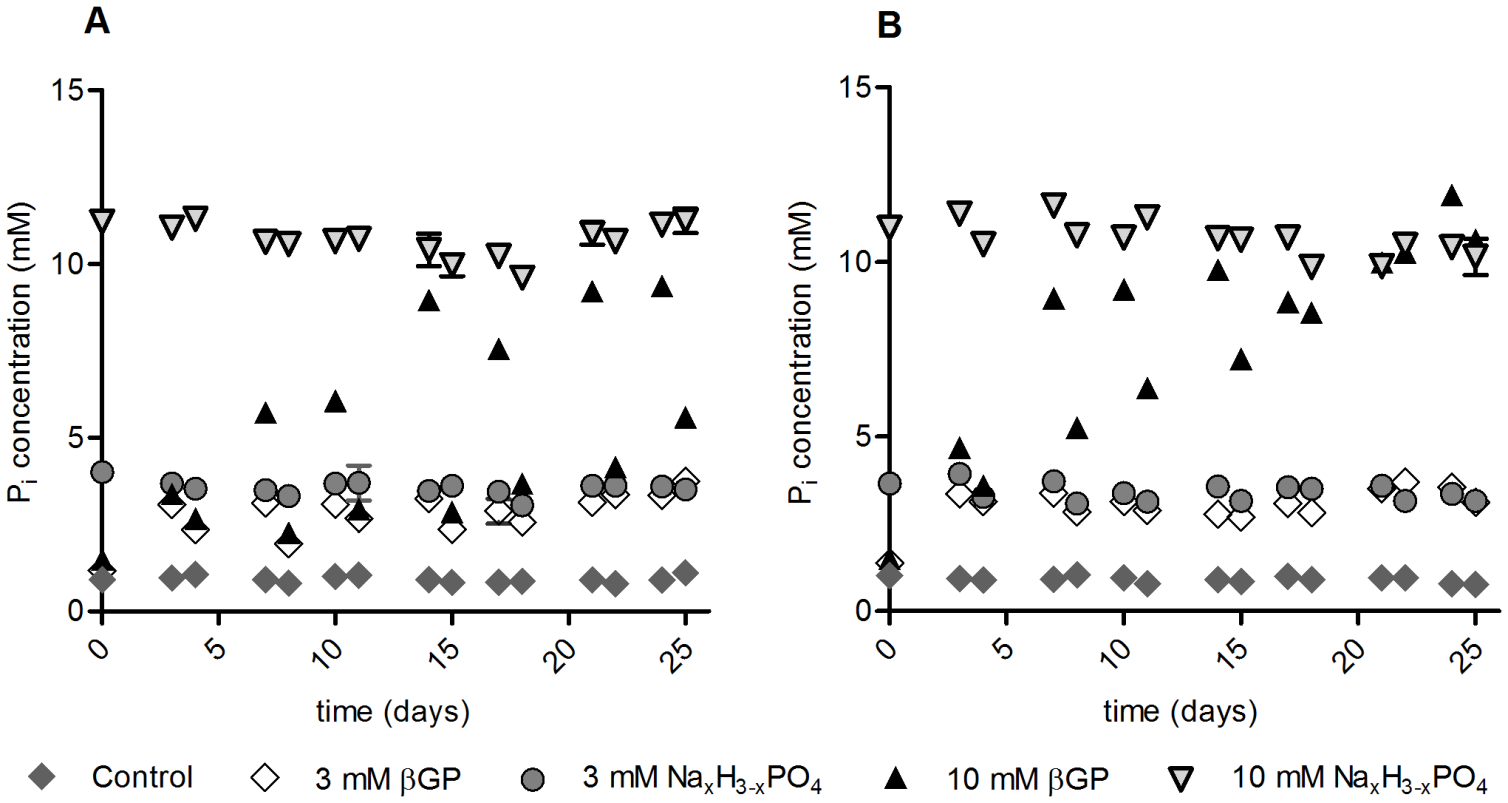
Free phosphate concentration. Concentration of free phosphate in the medium over the course of differentiation depending on the different phosphate sources shown for donors A and B respectively. Plotted as mean ± SD (small SD are not visible). A. Donor A (53-year old female). B. Donor B (47-year old male). Donors were chosen for relatively high difference in TNAP mRNA expression.

**Figure 2:**
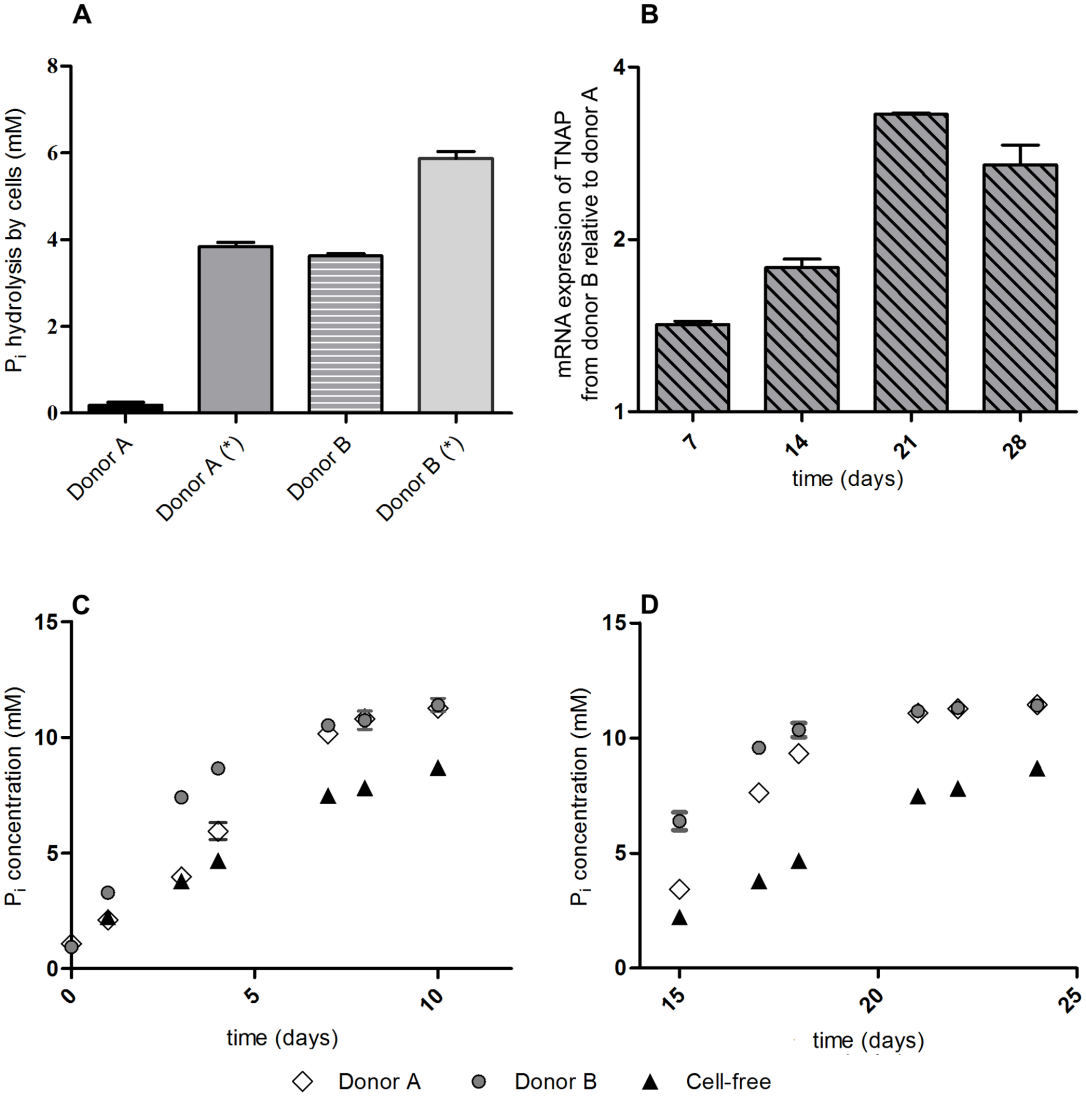
βGP hydrolysis and TNAP mRNA expression. A. Differences in phosphate hydrolysis over 3 days depending on the donor cells with (*) or without 14 days of pre-incubation in osteo-inductive medium containing 10 mM βGP with normal changes of medium. The free P_i_ concentration generated under cell-free conditions has been subtracted. Plotted is mean ± SD. B. Real-time PCR mRNA expression data of TNAP for donor B relative to donor A on days 7, 14, 21 and 28 of the osteogenic differentiation. C. Stability of βGP in the medium with cells and under cell-free conditions (no changes of medium) without pre-incubation of cells. Data plotted as mean ± SD (small SD not visible) D. Stability of βGP in the medium with cells and under cell-free conditions (no changes of medium) with 14 days of pre-incubation in osteo-inductive medium containing 10 mM βGP with normal changes of medium.

In order to induce osteoblast differentiation in mesenchymal stem cells, the growth medium needs to contain a certain level of free inorganic phosphate that allows for the mineralization to occur. We have evaluated the concentration of free P_i_ in osteogenic medium supplemented with different sources of phosphate over the course of differentiation (Figure 1A and B: Two representative donors are shown). With each change of medium the concentration of free P_i_ declines dramatically when the medium is supplemented with 10 mM β-glycerophosphate (and to a lesser degree when the medium is supplied with 3 mM β-glycerophosphate), whereas the P_i_ level remains fairly constant when phosphate is supplemented as Na_x_H_3-x_PO_4_. The rate with which β-glycerophosphate is hydrolyzed increases during the course of osteogenic differentiation. Also, an inter-donor difference in the rate of βGP hydrolysis is apparent (Figure 2, A: The same two representative donors are shown). Figure 2 C and D (without and with 14 days of pre-incubation of cells in osteo-inductive medium respectively) depict the hydrolysis of βGP over time when no changes of medium take place. Whereas during the first three days of osteogenic induction there is no substantial difference in the rate of βGP hydrolysis between donor A and a cell-free setting, the cells of donor B exert a profound influence on the rate of βGP hydrolysis (Figure 2 A, first versus third column). In order to assess whether this inter-donor difference in the βGP hydrolysis rate would dissipate during the course of osteogenic differentiation, the cells were pre-incubated for 14 days in osteogenic medium containing 10 mM βGP before repeating the above experiment (Figure 2D). After these 14 days of pre-incubation the rate of βGP hydrolysis in cells from donor A is comparable to the initial hydrolysis rate of the cells from donor B (Figure 2A, second versus fourth column), whereas the rate of βGP hydrolysis in cells from donor B is even higher than without the pre-incubation period. This means that in both cases βGP hydrolysis notably increases through prestimulation of the cells in osteogenic medium (Figure 2A: columns 2 and 4 compared to columns 1 and 3, respectively). This difference in phosphatase activity is reflected by the TNAP mRNA expression levels of donor B in comparison to donor A (Figure 2 B). Similar albeit less significant results were obtained for 3 mM βGP supplementation (data not shown). A possible explanation for the notable difference in Pi hydrolysis between donor A and B might be due to a huge difference in TNAP mRNA expression (more than 20 fold higher in donor B as compared to donor A, see Figure S1). We did not find a relationship between TNAP expression and donor age. The supplementation with 50 µM ascorbate-2-phosphate exerts no significant influence on the rate and level of phosphate release (data not shown).

### Phosphate Source and Concentration Influence Gene Expression of Commonly Used Osteogenic Marker Genes

**Figure 3:**
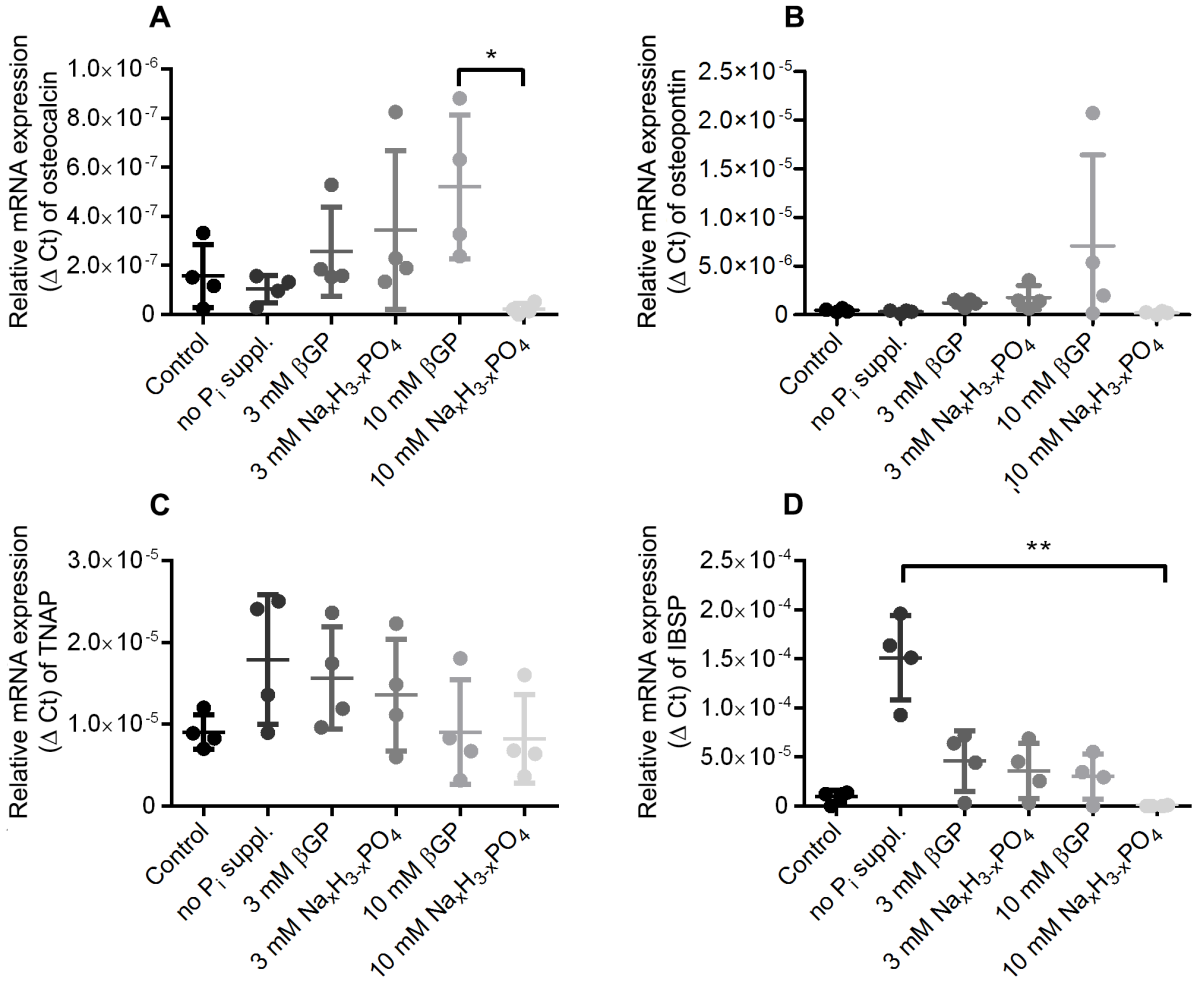
mRNA expression levels of osteogenic marker genes. mRNA expression levels of osteocalcin, osteopontin, TNAP and IBSP after 28 days of osteogenic differentiation depending on the phosphate source. The relative mRNA expression (Δ Ct) is plotted against the phosphate supplementation for five different genes involved in the formation of bone (n = 4). Plotted as mean ± SD.

It has been shown by Beck et al. that P_i_ is involved in the regulation of osteogenesis-related genes such as osteocalcin and osteopontin and that the influence of P_i_ on gene expression is concentration dependent [36]–[38]. We were able to confirm this result for osteocalcin for increasing concentrations of P_i_. Interestingly, we found that the use of 10 mM Na_x_H_3-x_PO_4_ seemed to have the opposite effect (Figure 3 A and B). On the contrary, TNAP and IBSP mRNA levels decrease with increasing P_i_ concentrations when cells are cultured in osteogenic induction medium (Figure 3 C and D). There are no substantial differences in gene expression between control and osteo-induced cells at days 7 and 14 for osteocalcin and osteopontin (data not shown).

In addition, we assessed the basal mRNA expression levels of osteocalcin, collagen Ia1 and TNAP among the cells from all 19 donors (including the 4 donors from the main study) during expansion of the MSC cultures at passage 2. There was a high inter-donor variability. There was a high inter-donor variability and therefore no significant differences were observed in the gene expression levels (Figure S1).

### FT-IR Results Demonstrate an Increase in the Mineral to Matrix Ratio with Increasing Levels of Free Phosphate and an Increase in Mineral Maturity for Low Concentrations of Free Phosphate

**Figure 4:**
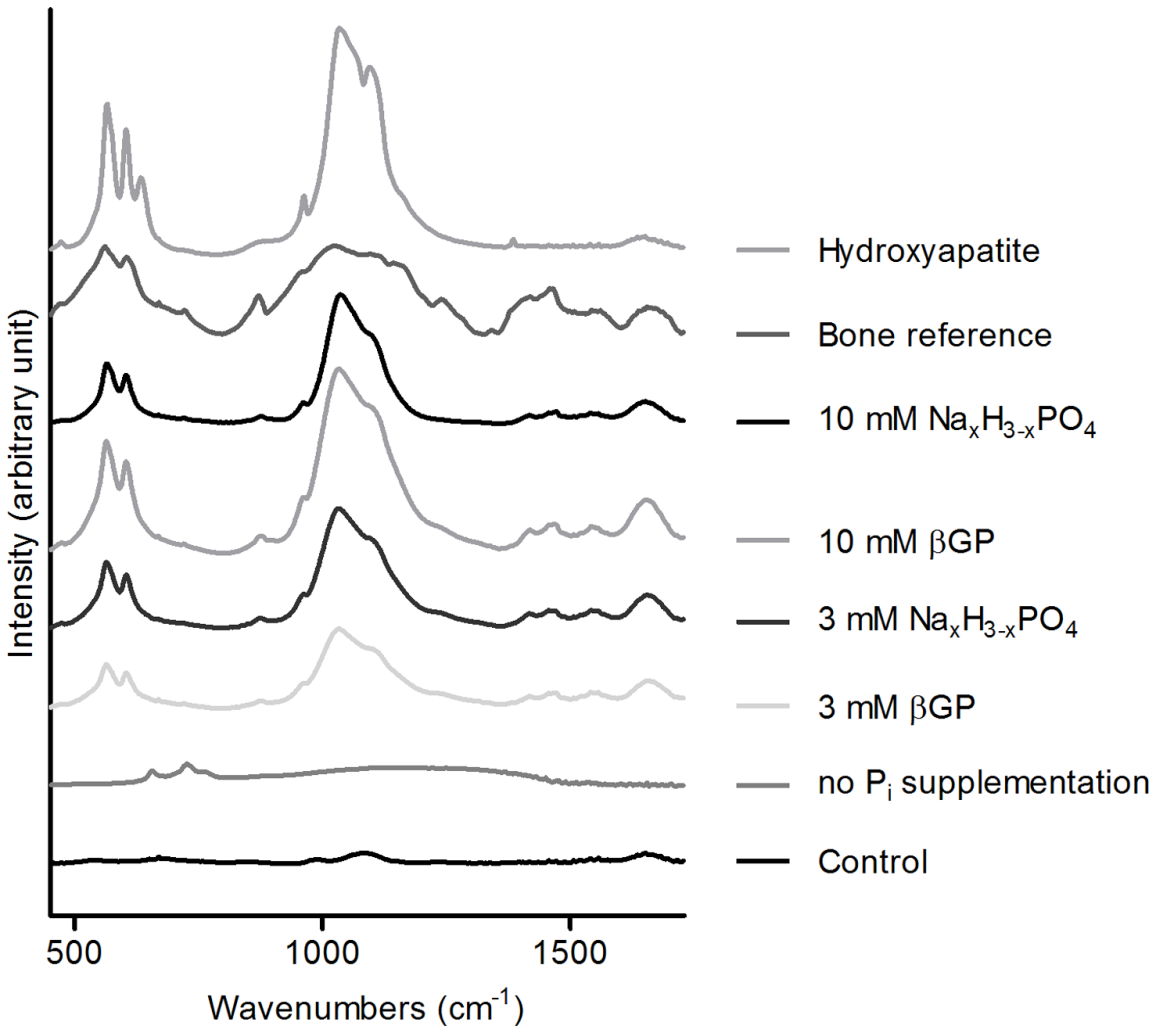
FT-IR spectra. FT-IR spectra (450–1750 cm^−1^) of samples cultured in different osteogenic induction or control media (n = 1) show the characteristic hydroxyapatite bands and the bands characteristic of the organic matrix of bone.

The FT-IR spectra of those samples which were supplemented with phosphate during osteogenic differentiation are all very similar both to the reference FT-IR spectrum of human bone and to spectra found in the literature (Figure 4). The spectra of the control and of the sample with 1 mM P_i_ show very weak bands characteristic for organic matrix components (amide I mode near 1650 cm^−1^ and amide II mode near 1550 cm^−1^ [39]) but no bands that hint at the deposition of calcium phosphate (β_1_,β_3_ phosphate region (900–1180 cm^−1^) [40]) and will thus not be considered further.

Apart from minor variances there are no qualitative differences between the IR spectra of the samples supplemented with 3 mM Na_x_H_3-x_PO_4_, 10 mM Na_x_H_3-x_PO_4_, 3 mM βGP, and 10 mM βGP. However, some aspects are worth noting: The mineral to matrix ratio (integrated peak area of phosphate bands divided by the integrated peak area of the amide I band) increases with rising levels of free phosphate (Table 1) [39]. Furthermore, the maturity indicated by the type of collagen crosslinks (determined by the area ratio of the pyridinoline (1660 cm^−1^) and the dehydrodihydroxylysinonorleucine (1690 cm^−1^) FT-IR bands) also increases from 3 mM βGP via 3 mM Na_x_H_3-x_PO_4_ to 10 mM βGP (Table 1). This ratio can be correlated to the ratio of nonreducible/reducible collagen cross-links in bone, which increases with bone maturation [41].

**Table 1:** Matrix and mineral characteristics derived from FT-IR data.

phosphate additive	Collagen crosslink(1690/1660 cm^−1^ ratio)	mineral maturity(1030/1110 cm^−1^ ratio)	mineral to matrix ratio(phosphate/amide I ratio)
**3 mM βGP**	1.75	3.27	5.99
**3 mM Na_x_H_3-x_PO_4_**	1.95	3.26	6.42
**10 mM βGP**	2.29	3.19	7.31
**10 mM Na_x_H_3-x_PO_4_**	2.03	2.91	9.21

Representative data of one donor for collagen crosslinks, mineral maturity and mineral to matrix ratios dependent on different phosphate additives in the osteo-induction medium.

In contrast, the mineral maturity (corresponding to the ratio of well-crystallized stoichiometric hydroxyapatite to nanocrystalline apatites, measured by the ratio of the 1030 cm^−1^ and the 1110 cm^−1^ IR bands (found in nanocrystalline apatites [42]) is highest for 3 mM βGP and 3 mM Na_x_H_3-x_PO_4_. The additives 10 mM Na_x_H_3-x_PO_4_ and 10 mM βGP lead to the formation of a higher percentage of nanocrystalline apatites (Table 1).

The characteristic peaks of hydroxyapatite are present in the spectra of all samples that were supplemented with additional phosphate. Apart from the bands attributable to hydroxyapatite (472, 561, 575, 601, 962, 1040, and 1092 cm^−1^) there are several bands present that hint at the presence of other calcium phosphates, such as dicalcium phosphate (892, 992, 1128, 1175, 1650 cm^−1^), dicalcium phosphate dihydrate (1070, 1132, 1646, 1730, and 2850 cm^−1^), and β-tricalcium phosphate (945, 972, 1041, 1094, and 1119 cm^−1^) [43]. The carbonate peak at 872 cm^−1^ is also represented in all spectra.

### XRD Results Demonstrate Formation of a Hydroxyapatite-like Matrix Independent of Phosphate Source and Concentration

**Figure 5:**
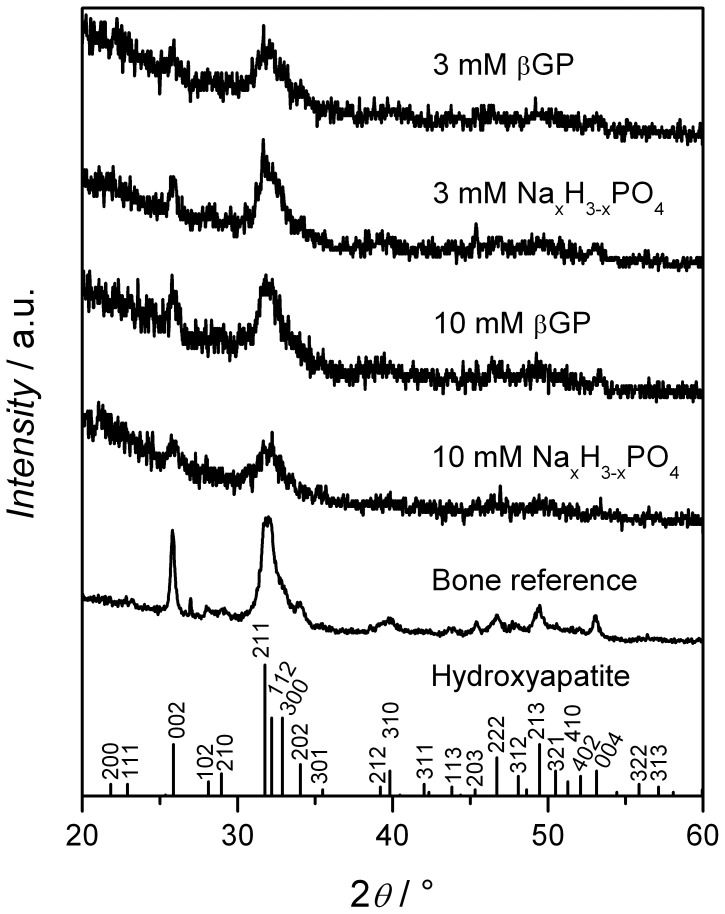
XRD patterns. XRD patterns of cells after osteogenic differentiation for 28 days supplemented with different sources of phosphate. Additionally, a fragment of human hip bone has been used as a reference sample of human bone. With second-derivation curve fitting analysis discrete reflections at 25.9 (002), 31.77 (211), 32.18 (112), 32.9 (300), 34.04 (202), and 39.79° (310) were detected for all samples supplemented with phosphate as well as for the bone reference sample. These reflections correspond to prominent peaks of hydroxyapatite [50].

Figure 5 shows representative XRD diffraction patterns of samples supplied with different sources of phosphate for differentiation. The underlying diffractions were curve-fitted. All phosphate supplements led to the formation of a mineralized matrix. The identified signals in the XRD patterns correspond to the reflections of hydroxyapatite and the diffraction patterns resemble that of human bone. The increased width of the signals may be due to a reduced crystallinity of the mineral formed or may indicate the formation of very small nanoparticles. No signals characteristic of monetite or octacalcium phosphate are found in any of the spectra. The XRD patterns of samples obtained with 10 mM βGP and 10 mM Na_x_H_3-x_PO_4_ show a weak broad signal between 30 and 32° 2*Θ* which hints at the presence of small amounts of amorphous calcium phosphate that may have formed by spontaneous precipitation due to the high concentration of phosphate ions.

### At Low Concentrations of Free Phosphate the Calcium to Phosphate Ratio is Similar to Bone

**Figure 6:**
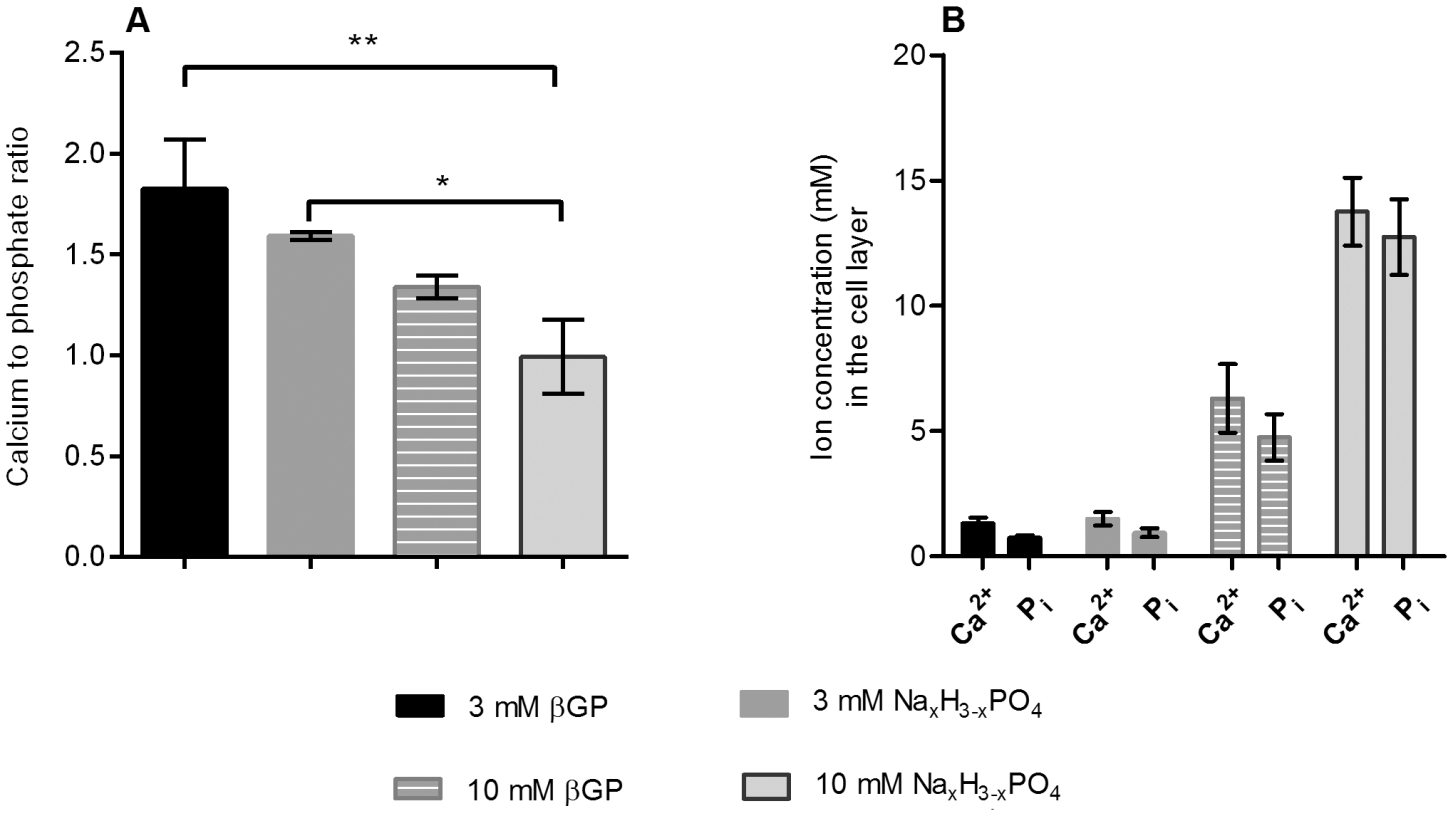
Calcium to phosphate ratios and ion concentration in the cell layer. A. Calcium to phosphate ratio. Calcium and phosphate ions were extracted from the cell layer of four different donors and quantified. Data are mean ± SD for n = 4 donors. B. Calcium and phosphate content of the cell layer after 28 days of osteogenic induction with different phosphate additives. Data are mean ± SD for n = 4 donors.

The calcium to phosphate ratios of the mineralized matrix as determined by the measurement of calcium and phosphate ion content of the cell layer for the samples obtained from cell cultures with different phosphate additives are shown in Figure 6 A. Samples prepared in the presence of 3 mM βGP and 3 mM Na_x_H_3-x_PO_4_ both show ratios that are close to the ratio observed in human bone (1.63). The ratio decreases with higher phosphate concentrations and is lowest for 10 mM Na_x_H_3-x_PO_4_ with a ratio around 1. There is an inverse relationship between the calcium to phosphate ratio and the mineral to matrix ratio that has been calculated from the FT-IR spectra. The amount of mineral formed increases with rising levels of free inorganic phosphate (Figure 6 B).

### Von Kossa- and Alizarin Red S Staining Confirm Mineralization of the Cell Layer

The *von Kossa* staining as an indicator of phosphates and the *Alizarin Red S* staining as an indicator of calcium ions in the cell layer were positive for all samples cultured in osteogenic induction medium except for the one not containing an additional source of inorganic phosphate (Figure S2).

### High Concentrations of Free Phosphate Diminish CICP Production

It is thought that mineralization can occur in association with collagen fibrils of the extracellular matrix or in connection with cell debris derived from apoptotic cells [44]. The synthesis of collagen is hence an important parameter of osteogenic differentiation and was measured in our experiments by enzyme-linked immunosorbent assay for the detection of the *collagen Type I C-terminal collagen propeptides,* which are cleaved from collagens during the maturation process. The release of the propeptides is proportional to the production rate of collagens.

**Figure 7:**
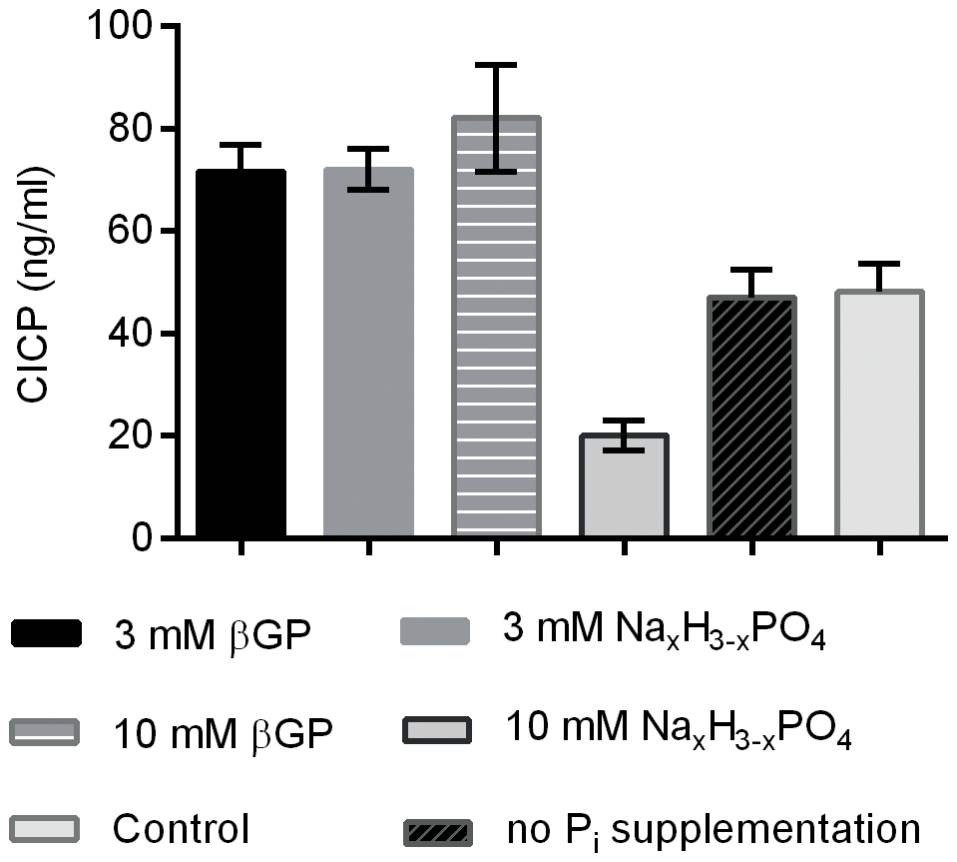
Rate of collagen synthesis. CICP assay of the supernatant during osteogenic induction. Data are mean of days 14 and 21± SD for n = 4 donors.

The addition of 3 mM βGP, 3 mM Na_x_H_3-x_PO_4_ or 10 mM βGP to the osteogenic induction medium leads to an enhanced collagen synthesis (Figure 7). Conversely, the addition of 10 mM Na_x_H_3-x_PO_4_ severely reduces the formation of new collagen. Dexamethasone and ascorbate-2-phosphate apparently exert no influence on the collagen production as the level of CICP in the sample containing no P_i_ supplementation is similar to the level in the control sample (Figure 7).

## Discussion

Originally, the rationale for using βGP as a source for phosphate ions was that “phosphate ions should be made available in places where alkaline phosphatase was present, thereby reducing the possibility of nonspecific mineral precipitation” [45]. This rationale would however only benefit the mineralization process if the hydrolysis of βGP was limited exclusively to the phosphatase activity of the cells. As βGP becomes hydrolyzed under cell-free conditions (Figure 2C, D, see cell-free conditions), there is no benefit in using βGP rather than Na_x_H_3-x_PO_4_. In light of the donor variability in βGP metabolism of human MSCs that is presented in this study it seems advisable to avoid the use of indirect phosphate sources if comparable conditions for *in vitro* osteogenesis are necessary for the experimental setup. We have screened MSCs from 19 different donors for their TNAP mRNA expression levels (Figure S1). Up to 53-fold differences in mRNA expression levels were found. The high inter-donor variability in TNAP expression levels and the resulting differences in the rate of phosphate release from βGP might account for the high inter-donor variability in osteocalcin and osteopontin gene expression. Furthermore the extreme fluctuations in free phosphate caused by the use of βGP are non-physiological and may exert an uncalculable influence on the differentiation process since it introduces a variable parameter dependent on the rate of P_i_ hydrolysis of the cells used. This is in line with the results of Khoshniat et al., who showed that the gene expression of both osteocalcin and osteopontin changes in response to an external phosphate stimulus as soon as 30 minutes after the addition of phosphate to the culture medium [46]. They also reported the up-regulation of osteocalcin and osteopontin in a P_i_ concentration-dependent manner [46], [47]. While our data does show a P_i_ concentration dependent up-regulation of osteocalcin and osteopontin, this holds true only within a certain concentration range. At 11 mM of free P_i_ (1 mM basal concentration in the medium and 10 mM added as Na_x_H_3-x_PO_4_) these two genes become down- rather than up-regulated. Therefore, a critical maximum free P_i_ concentration seems to exist, above which the gene expression of osteocalcin and osteopontin becomes repressed rather than activated.

The FT-IR data also show that mineral maturity suffers from more highly concentrated phosphate supplements. Apart from that, it can be concluded that with all phosphate supplements tested, the spectra are similar to the spectrum of a bone reference. The spectra from the different phosphate supplementations clearly demonstrate that the mineral formed is hydroxyapatite, as becomes apparent upon comparison with the FT-IR hydroxyapatite reference spectrum. Like the FT-IR spectra, the XRD patterns for all different phosphate supplementation groups are similar to each other as well as to the bone reference. The prominent reflections of hydroxyapatite are present in all patterns, and diffraction patterns typical of other calcium phosphate compounds such as octacalcium phosphate are absent. When comparing the diffraction patterns obtained from the groups with the higher phosphate concentrations 10 mM βGP and 10 mM Na_x_H_3-x_PO_4_ to those obtained with 3 mM βGP and 3 mM Na_x_H_3-x_PO_4_ the subtle broad peak characteristic of either partially amorphous material or of the formation of very small calcium phosphate nanoparticles becomes apparent, which shows that with 10 mM βGP and 10 mM Na_x_H_3-x_PO_4_, a certain degree of spontaneous precipitation takes place. This spontaneous precipitation of calcium phosphate also explains the decrease of the calcium to phosphate ratio of the mineralized matrix (Figure 6 A) with higher phosphate concentrations. The cause for the precipitation lies in the super-saturation of the differentiation media (Table 2). At a pH of 7.4, amorphous or only partially crystalline calcium phosphates precipitate when the solubility product of 5.5 mM^2^ is exceeded [18]. As proteoglycans have an inhibitory effect on this precipitation, the concentration product of Ca^2+^ and P_i_ can actually lie above this value [48], which accounts for the absence of amorphous calcium phosphate at the lower phosphate concentrations.

**Table 2:** Calcium phosphate ion products.

	Without P_i_ supplementation	3 mM Na_x_H_3-x_PO_4_/βGP	10 mM Na_x_H_3-x_PO_4_/βGP
Ca*P_i_ (mM^2^)	2.0	8.0	20.0

Calcium phosphate products in the different osteogenic induction media assuming 100% hydrolysis of βGP and 2 mM Ca^2+^ in the serum supplement (resulting in 2 mM Ca^2+^ in the basal medium).

As already mentioned in the introduction, the *von Kossa* or the *Alizarin Red S* staining methods are used to assess the mineralization of the samples treated to osteogenic induction. The former makes use of the fact that silver phosphate has a low solubility product: silver nitrate is added to the cell layer leading to yellow deposits of silver phosphate being formed when calcium phosphates are present. However, Ag^+^ ions can also react with carbonate ions to silver carbonate which precipitates as a white solid and, like silver phosphate in the presence of organic material, turns to black upon exposure to light. This makes it impossible to discriminate between phosphates and carbonates and can lead to a false positive staining pattern, thus making the *von Kossa* stain useful only when both staining and documentation procedures are performed in the absence of light. During *Alizarin Red S* staining the dye forms red chelate complexes with divalent cations, here Ca^2+^. *Alizarin Red S* is commonly used at an acidic pH around 4.5. Puchtler et al. have pointed out that the staining at a low pH will lead to diffusion artifacts. Furthermore, they have shown that *Alizarin Red S* will also form red chelates with cations of many other compounds [29]. Calcium-binding proteins and proteoglycans are also detected by this staining [49]. Therefore, this staining procedure should be performed at a pH of 9.5 and likewise be interpreted with care. In summary, histochemical staining is a limited substitute for physicochemical assays on the nature of the mineral formed [18]. The limitations to both the *von Kossa* and the *Alizarin Red S* staining methods call for a method that can prove the concomitant presence of calcium and phosphate ions in the cell layer and thus hint at a mineralized extracellular matrix typical for bone.

In the experiments performed by our group we found the quantification of calcium and phosphate ions extracted from the cell layer to be a fast and inexpensive alternative for the histological staining. The presence of precipitated calcium phosphate in the mineral formed was detected in our experiments by this method via the calcium to phosphate ratio. It must be noted however, that this method does not allow for the discrimination between ions from deposited calcium phosphate and ions that were bound to proteins.

When comparing the usefulness of XRD and FT-IR analyses to the ion quantification, it needs to be made clear that the calcium to phosphate ratio does not give direct information on the calcium phosphate compounds formed in the extracellular matrix during the differentiation process, but in the experiments performed here it correlates well with the information derived from the XRD patterns: ratios close to the ratio found in bone correlate with XRD patterns similar to XRD patterns found in bone and of hydroxyapatite. The spontaneously formed calcium phosphate precipitates received at higher phosphate levels could not be detected in the FT-IR spectra and only produced a weak broad peak in the XRD pattern, whereas they were clearly represented by a calcium to phosphate ratio lower than that found in human bone.

### Conclusions

Gene expression analysis alone is not a sufficient tool to assess *in vitro* osteogenic differentiation as the common marker genes osteocalcin and osteopontin are up-regulated by the addition of phosphate to the differentiation medium and thus do not represent reliable markers for the differentiation process itself, although their expression levels seem to increase with the amount of mineral deposited increasing (not true for 10 mM Na_x_H_3-x_PO_4_ as discussed above). The up-regulation of both TNAP and IBSP is not linked dose-dependently to the phosphate level present in the medium, and their expression does not correlate with the mineralization process.

When XRD analysis is not an option (due to inavailability or the rather large sample amounts needed), the quantification of the calcium and phosphate content of the cell layer should replace the classic histological *von Kossa* and *Alizarin Red S* staining as the histological methods for reasons given above. The quantification should be performed together with gene expression analysis of IBSP, TNAP, osteocalcin, and osteopontin.

The use of high phosphate concentrations to enhance the *in vitro* osteogenic differentiation should be avoided for several reasons. Firstly, high concentrations of free phosphate can have a deleterious effect on gene expression. Secondly, the use of 10 mM βGP leads to extreme non-physiological fluctuations of the extracellular phosphate level. As it is well known that the extracellular phosphate concentration directly influences transcription of several genes crucial for osteoblast development, such a fluctuation should be avoided. Lastly, we have shown that high concentrations of free phosphate in the differentiation medium lead to a lowered calcium to phosphate ratio in the cell layer, which is probably due to spontaneous precipitation of calcium phosphate.

In light of the results obtained by gene expression, XRD and FT-IR analysis as well as by calcium and phosphate ion concentration quantification, it seems advisable to use lower concentrations of phosphate, such as 3 mM Na_x_H_3-x_PO_4_ (resulting in a total P_i_ concentration of 4 mM) when working with human mesenchymal stem cells, as these supplements led to a mineralized matrix of good quality for all donor cells tested. Na_x_H_3-x_PO_4_ is superior to βGP in respect to providing an invariable source of free phosphate. The only disadvantage lies in the fact that a lot of data on *in vitro* osteogenic differentiation of human mesenchymal stem cells has been generated with 10 mM βGP as phosphate source, thus making it difficult to compare data to those studies. Nevertheless, we think it worth to consider a switch.

A detailed protocol for the in vitro osteogenic differentiation of human mesenchymal stem cells is given in the Supporting Information.

## Supporting Information

Figure S1 mRNA expression of osteogenic marker genes. Relative mRNA expression of osteocalcin, collagen Ia1 and TNAP from 19 different donors in passage two of *in vitro* culture. The four donors used in this project for the osteogenic differentiation tests are marked accordingly. Cells were harvested before reaching confluence.
(TIF)

Figure S2 Von Kossa and Alizarin Red S staining. Representative data from day 28 of the osteogenic differentiation for the different phosphate sources is shown (Alizarin Red S: left panel, von Kossa: right panel)
(TIF)

Protocol S1. (DOCX)
