# Supplementary figures and images for: The Phosphate Source Influences Gene Expression and Quality of Mineralization during In Vitro Osteogenic Differentiation of Human Mesenchymal Stem Cells
Source: PLoS One. 2013 Jun 18;8(6):e65943. doi: 10.1371/journal.pone.0065943 (PMC3688813; doi:10.1371/journal.pone.0065943)

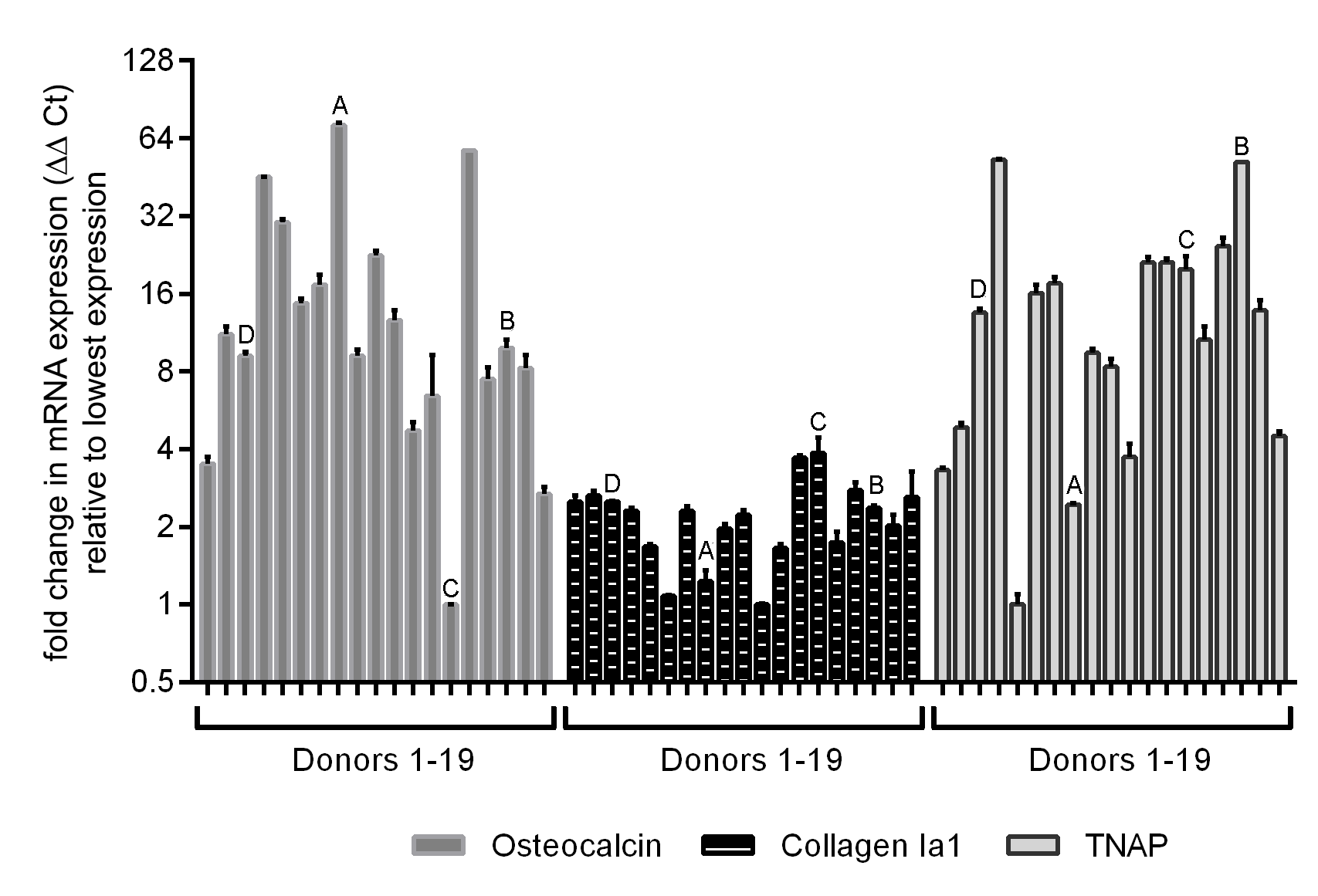

Supplement: Figure S1 — mRNA expression of osteogenic marker genes. Relative mRNA expression of osteocalcin, collagen Ia1 and TNAP from 19 different donors in passage two of in vitro culture. The four donors used in this project for the osteogenic differentiation tests are marked accordingly. Cells were harvested before reaching confluence. (TIF) [file pone.0065943.s001.tif]

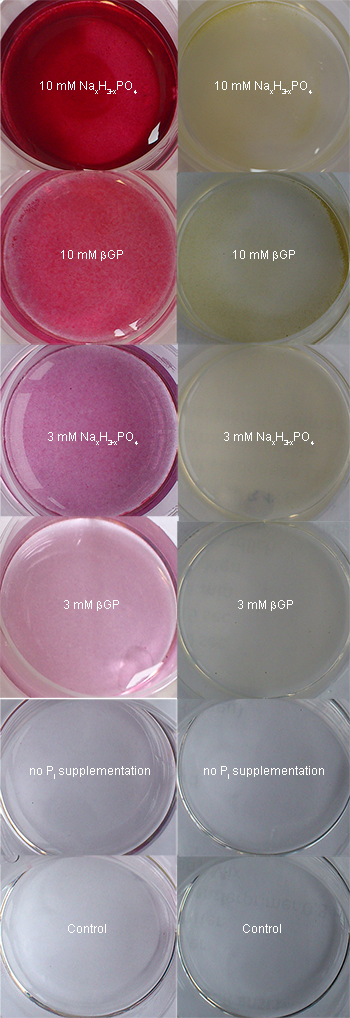

Supplement: Figure S2 — Von Kossa and Alizarin Red S staining. Representative data from day 28 of the osteogenic differentiation for the different phosphate sources is shown (Alizarin Red S: left panel, von Kossa: right panel) (TIF) [file pone.0065943.s002.tif]
