# Supplementary material for: The Phosphate Source Influences Gene Expression and Quality of Mineralization during In Vitro Osteogenic Differentiation of Human Mesenchymal Stem Cells
Source: PLoS One. 2013 Jun 18;8(6):e65943. doi: 10.1371/journal.pone.0065943 (PMC3688813; doi:10.1371/journal.pone.0065943)
Supplement: Protocol S1. — (DOCX) [file pone.0065943.s003.docx]

**Protocol S1.**

*Protocol S1 for in vitro osteogenic differentiation of human mesenchymal stem cells*

Human bone marrow-derived mesenchymal stem cells are best suited for *in vitro* osteogenic induction between passages 3 and 8, since contaminating hematopoietic cells may be present at passages 1 and 2 and prolonged *in vitro* cultivation decreases the differentiation potential. Seeding cells in one well of a 6-well culture plate (9.6 cm² growth area) is sufficient for RNA isolation, subsequent cDNA synthesis, and gene expression analysis. The same holds true for calcium and phosphate ion quantification of the cell layer. For FT-IR or XRD analysis a cell culture flask with 75 cm² or more should be used to acquire enough material. Cells are seeded at a density of 5000 cells per cm² and grown until confluence in MSC medium (see Materials and Methods section for composition), which is then replaced by osteogenic induction medium.

1. Culture the MSCs on non-coated cell culture flasks until enough cells are available for the osteogenic induction assay. Always passage cells before more than 75% confluence is reached (that way the cells retain their multilineage potential longer).
2. Once you have enough cells for the experiment, prepare the amount of 6-well dishes (and 75 cm² flasks if you want to harvest cells for FT-IR and XRD analyses) needed by coating them with 2% gelatin B (necessary to avoid detachment of cells during the osteo‑induction):
3. Pipette 1 ml of sterile 2% gelatin B into each well of the 6-well dishes (and 7 ml of sterile 2% gelatin B into each 75 cm² cell culture flask). Incubate for 30 min at 37 °C, remove the gelatin and wash three times with appropriate volumes of PBS. Leave the coated wells covered with PBS to keep them from drying out until shortly before plating cells. They can be stored for several days at room temperature or at 4 °C.
4. Remove the MSC medium from the cells. Wash once with an appropriate amount of PBS and detach the cells from the flasks by adding 0.04 ml per cm² of 0.025% trypsin/EDTA.
5. Incubate for 2 min at 37 °C, then clap your flat hand to the side of the culture flask several times and check under the microscope whether all cells are floating. If so, continue to step 6. If not, repeat the incubation and clapping procedures until no adherent cells are left.
6. Suspend the cells in MSC medium (use at least three times the volume of trypsin/EDTA) and transfer them into a sterile tube.
7. Plate the cells at a density of 5000 cells per cm². When using 6-well plates: use enough medium as the cells may die if too little medium is used upon plating (at least 3 ml of medium for a well of 9.6 cm²).
8. Let the cells grow to confluence.
9. Upon reaching confluence, begin the osteo-induction:
10. Prepare a sufficient amount of osteo-inductive medium composed of DMEM with 1 g/l D-Glucose, 10% (v/v) FCS, 2% HEPES, 1% penicillin and streptomycin, 100 nM dexamethasone, 50 µM sodium ascorbate‑2‑phosphate and 3 mM Na_x_H_3‑x_PO_4_ (dexamethasone and sodium ascorbate-2-phosphate can be kept as 100 x stock solutions at ‑ 20 °C, Na_x_H_3‑x_PO_4_ can be stored as 100 x stock solution at room temperature). For 3 mM Na_x_H_3‑x_PO_4_ prepare stock solutions of both 100 mM Na_2_HPO_4_ and NaH_2_PO_4_, then use the Henderson-Hasselbach equation to calculate the amount of 100 mM Na_2_HPO_4_ and 100 mM NaH_2_PO_4_ needed to achieve a pH of 7.4 and a phosphate concentration of 3 mM for a certain volume. Check the pH and adjust with HCl and NaOH if necessary.
11. Remove the MSC medium, wash the cells in an appropriate amount of PBS and add 3 ml of osteo-inductive medium. Keep in mind that you also need a control that is cultured in MSC medium without FGF-2.
12. Change medium twice a week. Medium should be prepared fresh once a week.
13. For RNA isolation remove the medium, wash once with an appropriate amount of PBS and add 1 ml Trizol per well to the cells (or proceed as required by RNA isolation kit) and pipette up and down several times with a Pasteur pipette. Transfer the Trizol sample to an 1.5 ml reaction tube and store at – 80 °C until further processing. The authors deem the up-regulation of osteocalcin, osteopontin and IBSP to be the best suited genes for analysis of osteogenesis-dependent gene expression.
14. In order to quantify calcium and phosphate ions from the cell layer remove the medium, wash three times with an appropriate amount of TBS and fix the cells in 4% PFA (needs to be either freshly prepared or stored at – 20 °C until use; prepare in TBS). Incubate 1 ml per well (9.6 cm²) for 30 min at room temperature. Then remove the PFA completely and wash three times with an appropriate amount of TBS (do not use PBS). Add 0.5 ml of 1 M HCl and remove the cells by using a cell scraper. Transfer the suspension into a 1.5 ml reaction tube. Flush the well with another 0.5 ml of 1 M HCl and transfer to the reaction tube as well. Incubate overnight at 37 °C at 800 rpm. Centrifuge for 15 min at 21.000 x g in order to sediment the cell debris. Transfer the supernatant to a fresh reaction tube and store at – 20 °C until quantification of calcium and phosphate ions. Start with a dilution of 1 : 100.
15. The osteo-induction period should be 28 days.
